# ATR-FTIR Biosensors for Antibody Detection and Analysis

**DOI:** 10.3390/ijms231911895

**Published:** 2022-10-07

**Authors:** Olivier Suys, Allison Derenne, Erik Goormaghtigh

**Affiliations:** Center for Structural Biology and Bioinformatics, Laboratory for the Structure and Function of Biological Membranes, Campus Plaine CP206/02, Université Libre de Bruxelles, B1050 Brussels, Belgium

**Keywords:** FTIR spectroscopy, ATR, biosensors, antibody detection, SAM grafting, germanium, immunoglobulin-binding protein

## Abstract

Quality control of drug products is of paramount importance in the pharmaceutical world. It ensures product safety, efficiency, and consistency. In the case of complex biomolecules such as therapeutic proteins, small variations in bioprocess parameters can induce substantial variations in terms of structure, impacting the drug product quality. Conditions for obtaining highly reproducible grafting of 11-mercaptoundecanoic acid were determined. On that basis, we developed an easy-to-use, cost effective, and timesaving biosensor based on ATR-FTIR spectroscopy able to detect immunoglobulins during their production. A germanium crystal, used as an internal reflection element (IRE) for FTIR spectroscopy, was covalently coated with immunoglobulin-binding proteins. This thereby functionalized surface could bind only immunoglobulins present in complex media such as culture media or biopharmaceutical products. The potential subsequent analysis of their structure by ATR-FTIR spectroscopy makes this biosensor a powerful tool to monitor the production of biotherapeutics and assess important critical quality attributes (CQAs) such as high-order structure and aggregation level.

## 1. Introduction

Proteins are widely used as biotherapeutics (e.g., antibodies, hormones, and enzymes). They form the fastest-growing class of therapeutics and are applied in a large diversity of disorders such as cancers, autoimmune diseases, or metabolic illnesses [1]. Convenient monitoring of protein critical quality attributes (CQA’s) such as conformation or glycosylation level in the course of production has become essential but not sufficient. Indeed, during protein production, storage, transport, and delivery to the patient, loss of native structure is common. Protein aggregation is part of the structural changes that can be observed and is particularly critical for proteins used as therapeutics [2]. Such structural changes are accompanied by definite changes in the protein secondary structure [3,4]. Ideally, a biosensor should be not only be able to assess the presence of a particular protein in a complex sol ution but should also evaluate the CQAs of the detected protein.

**Figure 1:**
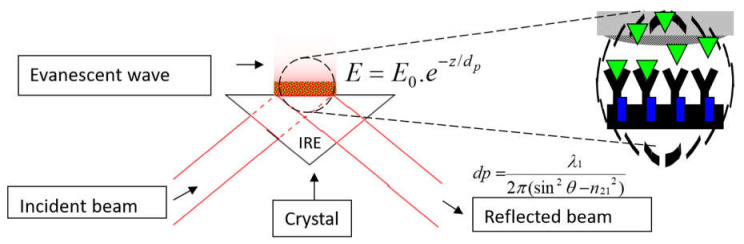
Schematic representation of the ATR-FTIR setup. The incident beam is totally reflected on the surface of the internal reflection element (IRE), here a germanium trapezoidal prism, but an evanescent field **E** exists in the outer medium over a distance in the range of a few µm. Its intensity decays exponentially with the distance *z* from the reflecting interface where its value is **E_0_**. The characteristic penetration depth d_p_ depends on the incidence angle θ, the ratio n_21_ between the refractive index within the germanium n_1_ and outside n_2_, n_21_ = n_2_/n_1_, and the wavelength λ, with λ_1_ = λ/n_1_.

Fourier transform infrared (FTIR) spectroscopy is a flexible spectroscopic approach that can report protein secondary structure [5,6,7,8,9,10,11,12,13,14,15] and simultaneously protein glycan content [16] or lipid content [17,18]. One challenge is to carry out accurate measurements in an aqueous environment. Indeed, the protein amide ν(C=O) band, called Amide I, is found between 1700 and 1600 cm^−1^ and the amide δ(N-H) band called Amide II is found between 1600 and 1500 cm^−1^. They are both sensitive to protein secondary structure but strongly overlapped by the water δ(OH) absorbance band [19]. The use of attenuated total reflection (ATR) FTIR spectroscopy allows the limitation of the optical path length in the aqueous medium and therefore prevents saturation of the detector. In ATR-FTIR, the infrared beam is reflected inside an internal reflection element (IRE) such as a germanium or diamond crystal, and absorption by the sample takes place beyond the reflection point where the so-called evanescent wave penetrates in the rarer medium, usually by less than a few µm [20]. ATR-FTIR is therefore one of the most powerful methods to record FTIR spectra of biological molecules [20,21,22]. Another interesting aspect of ATR-FTIR is that, if a receptor is grafted on the germanium surface, any molecule that binds to the receptor will enter the region where the evanescent wave is intense and will generate a spectrum while the molecules present in the bulk are too diluted to generate a significant absorbance. ATR-FTIR is then perfectly fit for building biosensors [23,24,25]. The basic principles that make ATR-FTIR suitable for building biosensors are illustrated in Figure 1.

Because of its high refractive index, chemical properties, and large spectral window in the mid-IR, germanium is often preferred over other materials as IRE [23,24,25].

The second challenge therefore consists in both attaching a receptor, most often an antibody, on the germanium surface, and preventing non-specific adsorption. Attachment between a protein onto a surface by passive adsorption relies on non-covalent interactions. While this strategy requires no specialized chemistry and is easy to use, limitations include frequent partial protein denaturation with loss of activity and interaction capability. Covalent grafting on a surface is often preferred to obtain surface functionalization that is reliable, reproducible, and delivers robust interfaces which are “protein-friendly” and prevents non-specific adsorption. While protein immobilization by alkanethiols is well-established on gold surfaces, the chemistry of the germanium surface is still under development. Initially, binding through silane groups was developed [26] and successfully applied to several cases [24,27]. Yet, stability remained an issue, and new chemistry based on thiol reactivity was developed [25]. These sensors were shown to be quite efficient at detecting markers of Alzheimer’s disease in complex media [28]. Interestingly, the secondary structure of the amyloid beta-peptide could be assessed in complex fluids [29], demonstrating the capability of ATR-FTIR-based biosensors to detect a peptide and determine its secondary structure [30,31]. Other promising grafting strategies based on calixarene are currently under development [32,33].

In the present work, we developed the experimental conditions for achieving a highly reproducible grafting procedure based on 11-mercaptoundecanoic acid to attach immunoglobulin-binding proteins (i.e., protein A, protein G, or protein A/G) onto a germanium surface. Protein A can capture monoclonal antibodies (mAbs) efficiently and is often used for affinity chromatography. It can be recycled by lowering the pH even though binding capacity decreases with the number of cycles [34]. We show here that an ATR-FTIR biosensor can specifically detect mAbs in a culture medium and that information on the conformation of the mAbs can be potentially obtained. This observation opens the way to control the quality of antibodies during the culture evolution without removing any excipients or applying any buffer exchange.

## 2. Results

**Figure 2:**
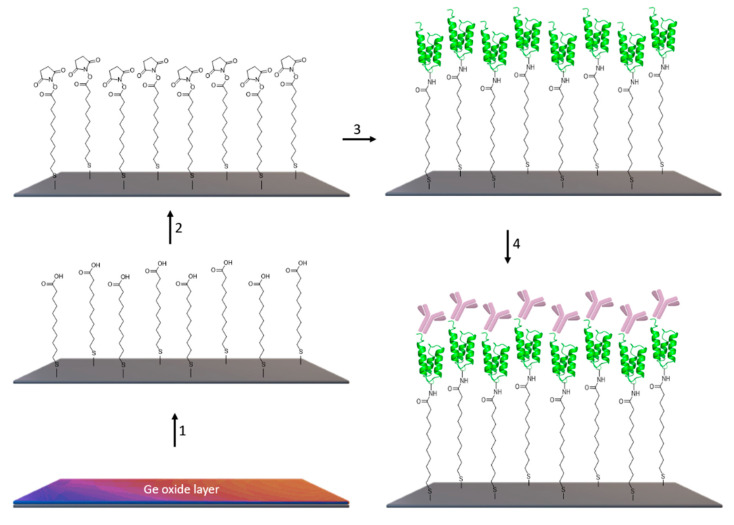
Schematic sketch of the assembly of the biosensor. Step 1: Removal of the germanium oxide layer and functionalization by 11-mercapto-undecanoic acid SAM, step 2: EDC/NHS carboxylate activation, step 3: immunoglobulin-binding protein immobilization (e.g., protein A), step 4: immunoglobulin binding onto protein A.

**Figure 3:**
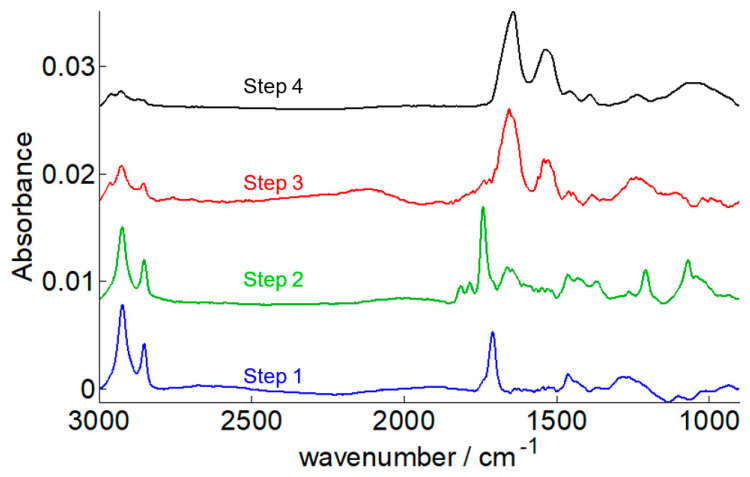
ATR-FTIR spectra obtained at different steps of the sensor building. Step 1: Removal of the germanium oxide layer and 11-mercapto-undecanoic acid SAM functionalization in blue, step 2: EDC/NHS carboxylate activation in green, step 3: immunoglobulin-binding protein A immobilization in red (after ethanolamine quenching of the remaining NHS-ester functions), and step 4: immunoglobulin binding onto protein A in black. The background of each spectrum above is the spectrum of a clean Ge except for step 4 for which the background is the protein A immobilization in red (step 3). Spectra have been offset for the sake of the clarity.

The construction of the sensor is schematically presented in Figure 2. One of the interesting advantages of the ATR-FTIR method is that, without any labeling, the attachment of these molecules used to build the sensor attaching onto the surface of the IRE can be monitored through their specific absorption bands (Figure 3), providing convenient monitoring of the construction of the sensor.

Removal of the oxide layer is the first step to achieve before grafting a molecule on the germanium surface. As germanium oxide is not as stable as silicon oxide, the germanium oxide layer can be easily removed using water. Yet, unlike alcohols, water is not a suitable solvent for alkanethiol. Germanium oxide removal and thiol SAM preparation were thus carried out in a single step using 11-mercaptoundecanoic acid (MUA) dissolved in a 1/1 water/isopropanol (IPA) solution heated at 60 °C. A water/alcohol mixture can indeed dissolve both the germanium oxide and alkanethiols. In such condition, coverage reaches a maximum of 50% when the experiment is performed at room temperature while increasing the temperature to 60 °C leads to 100% coverage [35]. While testing several grafting strategies, we found out that the combination of the one-step oxide removal and thiol grafting based on the use of water/isopropanol and of the 60 °C temperature was key to achieve robust and reproducible biosensors. The FTIR spectrum of the 11-mercaptoundecanoic acid grafted onto the surface is characterized by three well-defined main bands: 2924 cm^−1^ ν_as_(CH_2_), 2853 cm^−1^ ν_s_(CH_2_) and 1711 cm^−1^ ν(C=O) as illustrated by the blue spectrum in Figure 3.

Comparison with a control built in the absence of MUA (Figure S1) and comparison with the grafting of fully deuterated MUA (D_25_ MUA) (Figure S2) with ν_as_(C^2^H_2_) at 2196 cm^−1^ and ν_s_(C^2^H_2_) at 2095 cm^−1^ confirms that MUA did bind to the germanium surface.

The activation step can be monitored in FTIR spectroscopy by the disappearance of the MUA-COOH stretching at 1711 cm^−1^ and the appearance of three bands assigned to the formation of the ester bond, -COO-NHS (1816 cm^−1^, 1788 cm^−1,^ and 1741 cm^−1^) and of specific NHS bands at 1207 cm^−1^ and 1068 cm^−1^ (Figure 3—green spectrum). Spectra of the pure NHS and the NHS ester are reported in Figure S3.

**Figure 4:**
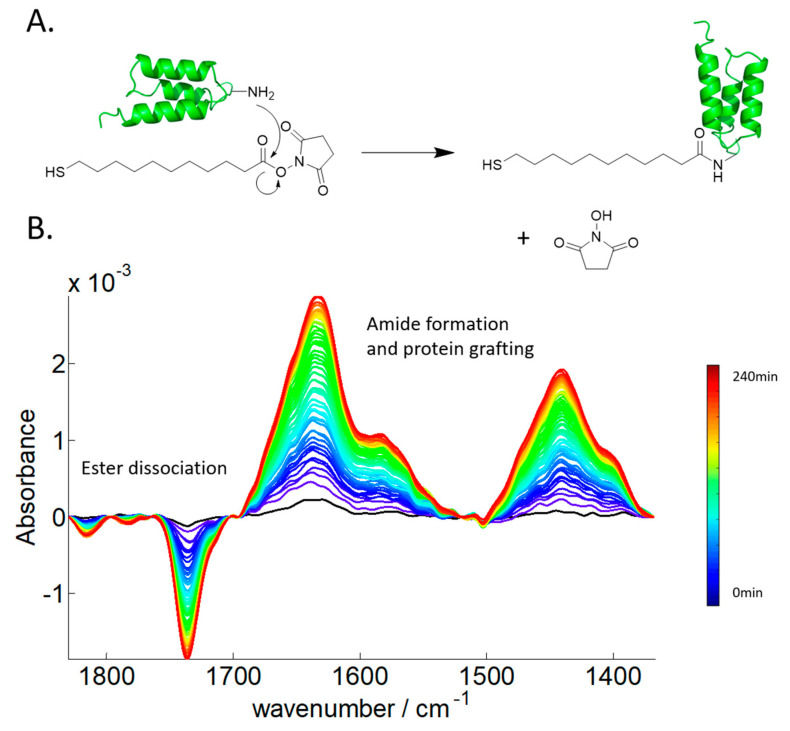
(**A**). Aminolysis reaction between the MUA-NHS ester and protein A. The nucleophilic attack of the protein via the primary amines is possible because the poor hydroxyl leaving group has been previously activated to produce a good leaving group, the NHS. (**B**). Evolution as a function of the time of the FTIR spectrum of the MUA-NHS surface placed in the presence of protein A (1 mg/mL in 0.1 M PBS buffer at pH 7.4 dissolved in ^2^H_2_O). At time = 0 min, the spectrum recorded is used as the background and is subtracted from all subsequent spectra. Short incubation time spectra are plotted in blue and the longest incubation times are in the red as indicated by the color bar placed on the right-hand side of the figure. Total duration of the kinetics is 3 h, and a spectrum is recorded every 3 min (60 spectra in total).

Once activated, any protein containing lysine side chain available on its surface can potentially bind onto the crystal surface. A new amide bond is formed between the lysine, containing the primary amine, and the activated ester, with the release of the NHS molecule as schematically depicted in Figure 4A. This aminolysis reaction can be monitored online by FTIR spectroscopy (Figure 4B). Clearly, both the new amide bands and the loss of the ester band of the NHS are visible on the spectra. As the binding has been recorded in heavy water (^2^H_2_O) to avoid overlap with the ^1^H_2_O contribution in the amide spectral region, the amide I band is shifted by 10 cm^−1^ to lower wavenumbers and amide II intensity is reduced while a new band called amide II’ appears at 1440 cm^−1^ [36,37,38,39,40]. Indeed, it is well-known that in amide, I band, hydrogen-deuterium exchange (HDX) induces a slight shift by 5–10 cm^−1^ to lower wavenumbers upon N-H deuteration [6,40,41]. Exchange of the amide protons occurs at a rate that depends on protein structure and stability as described elsewhere [42,43,44,45]. Figure 4B presents the spectrum of the grafting of partially deuterated protein A onto the functionalized germanium surface as indicated by a small residual amide II contribution around 1560 cm^−1^. Spectra were recorded every 3 min for 3 h, resulting in 60 spectra.

**Figure 5:**
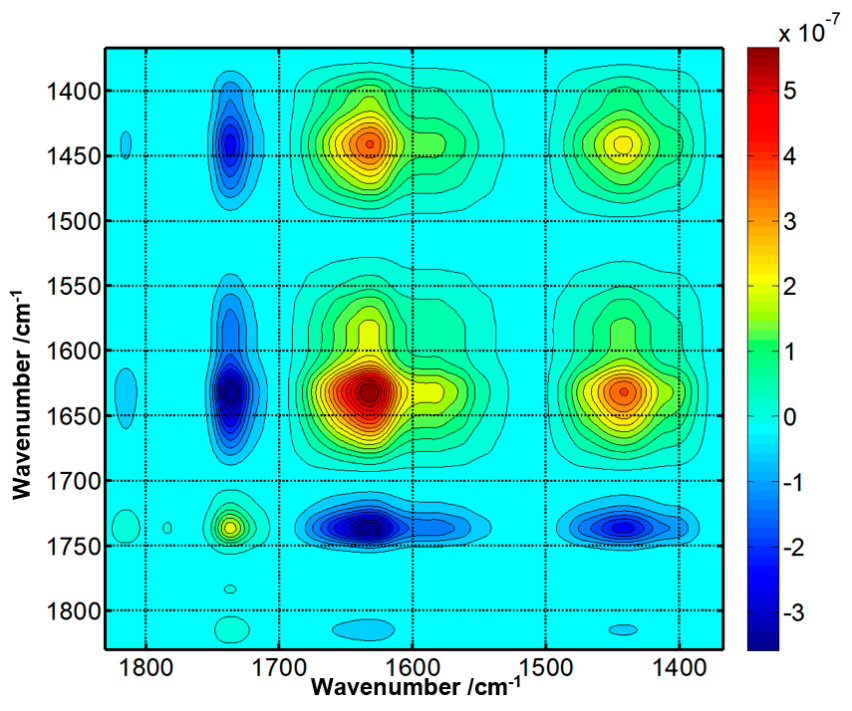
Magnitude of the covariance between the different wavenumbers during the grafting of protein A. The disappearance of the NHS ester at 1741 cm^−1^ is significantly and negatively correlated with the appearance of the amide bands at 1639 cm^−1^ and 1440 cm^−1^. Correlation coefficient between 1741 cm^−1^ and 1639 cm^−1^: −0.99, corresponding *p*-value 1.83 10^−50^. Correlation coefficient between 1741 cm^−1^ and 1440 cm^−1^: −0.9956, corresponding *p*-value 1.85 10^−61^. Correlation coefficient between 1639 cm^−1^ and 1440 cm^−1^: +0.9967, corresponding *p*-value: 4.72 10^−65^.

To assess the robustness of the method, proteins with different secondary structures (bovine serum albumin, avidin, and protein G) have also been grafted. As mentioned earlier, FTIR spectroscopy can estimate the secondary structure content of a protein after analysis of the shape and position of the amide bands. BSA is a protein containing mostly alpha-helices while avidin is mainly composed of beta-sheets. Immunoglobulin-binding protein G includes a mix of both structures. The analysis of grafting kinetics (Figures S4, S6 and S8) suggests that the method is robust as it works as efficiently for the three proteins. Furthermore, the amide I position reported in Figure S9 agrees with the secondary structure type of each protein, indicating that grafting per se does not induce significant structural changes. This is important as the attached proteins must retain their native structure to fulfill their functions, i.e., in the present case the binding of immunoglobulins. As proteins contained a mix of deuterated and undeuterated amide groups, quantitative determination of the secondary structure was not attempted. All immunoglobulin-binding proteins tested, protein A, protein G, and protein A/G have successfully been grafted. Interestingly, a correlation analysis indicates that the decay of the ester band at 1741 cm^−1^ resulting from the release of the NHS molecules is very well correlated with the appearance of the amide band at 1639 cm^−1^ (correlation coefficient R = −0.99) for protein A grafting (Figure 5), demonstrating that the two events progress at the same pace, though in the opposite direction. The same results have been obtained with BSA and avidin (Figures S5 and S7). This strongly suggests the occurrence of an aminolysis reaction and rules out a hydrolysis of the NHS ester accompanied by non-specific protein adsorption, as the two reactions would proceed at different paces.

Before measuring antibody binding onto the Protein A coated germanium, ethanolamine was used to remove unreacted NHS ester. This step is necessary to avoid unspecific protein grafting to the surface. The total disappearance of the peaks assigned to the NHS ester can be observed. Importantly, it is accompanied by no shift of the amide I band of the grafted protein A, confirming the absence of structural changes in the latter protein (red spectrum in Figure 3).

When the biosensor built with protein A was exposed to a complex medium, here a culture medium at the end of the culture (day 16), only antibodies bind to the surface. No significant binding occurred when protein A was replaced by BSA (Figure S10), and no significant binding was recorded when protein A was exposed to a culture medium taken before adalimumab molecules were expressed (day 0) (Figure S11). By using the FTIR spectrum obtained in the previous step (protein A grafted onto the surface) as the background, one thus obtained only the spectrum of the antibody, here adalimumab (Figures S10 and S11 and black line on Figure 3).

**Figure 6:**
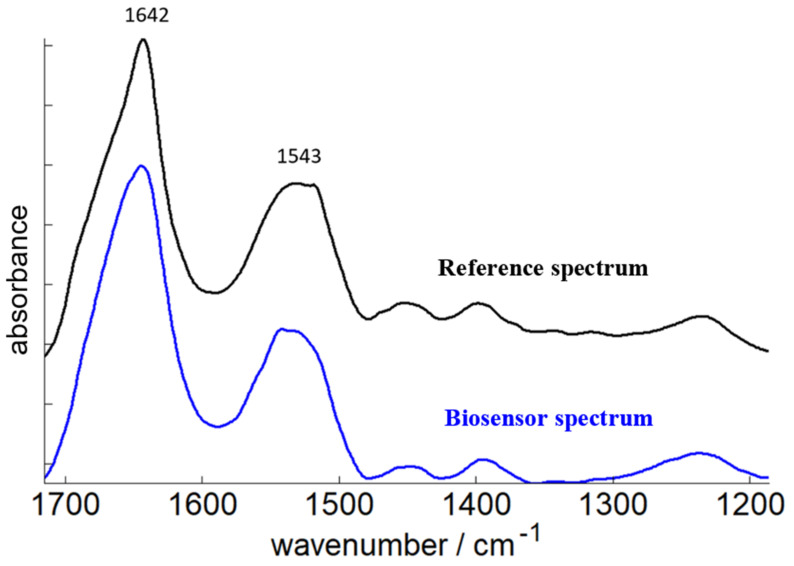
Comparison of purified adalimumab FTIR spectra gathered either by the developed biosensor or by pharmaceutical grade purification method from day 16 culture medium sample. The blue line corresponds to the spectrum of the antibody obtained via the biosensor exposed directly to the culture medium containing the mAb and the black line corresponds to the purified adalimumab reference followed by a desalting step by size exclusions column.

This spectrum obtained by the biosensor (blue line on Figure 6) was compared with the spectrum of purified adalimumab from the same culture medium sample (see Methods) (Figure 6 black line).

Before recording the reference spectrum on the diamond crystal, a desalting step (see Methods) was required to eliminate buffer and salt molecules that may interfere with the spectrum of the antibody. The desalting step was performed using size exclusions columns. This time-consuming step was not necessary with the biosensor since only the components grafted on the surface generated a significant FTIR signal.

The two spectra are very similar. The position of the maxima is almost identical for both the Amide I (1642 cm^−1^) and the Amide II (1543 cm^−1^). These wavenumbers are consistent with the antibody β sheet-rich secondary structure. This result underlines that the spectrum of the antibody recorded with the biosensor is exploitable in the spectral region corresponding to protein absorption. The analysis of high order structure and the aggregation level as well as stability studies are thus possible using the device developed in the present work.

## 3. Discussion

The development of biosensors presents a large interest and has become a fast-growing field of research in the past decades mainly because of their practicality. While the principle of ATR-FTIR biosensor has been established for a long time [26], grafting on germanium remained an issue. Among the different materials used for the ATR IRE, only silicon and germanium can provide adequate chemistry for attaching molecules. The higher refractive index of germanium and its wider spectral window make germanium preferable. Indeed, the glycan bands near 1000 cm^−1^ can be analyzed with a germanium IRE but not with a silicon IRE. As described in the introduction, the yield and stability of the grafted molecules on germanium has long remained a major issue for the application of ATR-FTIR biosensors. New types of grafting are still under development to overcome the problem [32,33].

This work uses a simple and robust method for surface protein grafting. A key feature of the work described in the present paper is that cleaned surfaces were immersed in a 50 mM MUA water/isopropanol 50/50 solution overnight at 60 °C. The temperature of 60 °C was found to be critical for obtaining optimal grafting in terms of density and stability. For the next step, the use of chemical groups that react with primary amines (i.e., lysines) is one of the most versatile techniques for protein conjugation [46]. As the hydroxyl group of MUA is a poor leaving group, it needs to be activated to react with proteins. One of the best-known methods consists in forming an N-hydroxysuccinimide (NHS) ester with the carboxyl group of MUA using EDC and NHS. With NHS being a good leaving group, the reaction is now conducive to nucleophilic attack by primary amines present in the protein. In the course of sensor building, it was observed that the correlation coefficient between the ester disappearance and the amide formation was very close to 1.00, suggesting that we are not observing, on the one hand, NHS ester hydrolysis followed, on the other hand, by non-specific protein adsorption, as both processes are likely to proceed at different paces. This strongly suggests a specific covalent NHS ester aminolysis by the primary amines of the protein. This type of insight into the reactions involved in sensor building is one of the advantages of the ATR-FTIR sensor.

Another advantage of this ATR-FTIR sensor is that it provides not only a reading of the binding, but also insight into the FTIR spectrum of the bound molecules. When proteins are concerned, as this FTIR spectrum is sensitive to their secondary structure, information on the secondary structure could be obtained. Beside the assessment of the native secondary structure, the intense signal of the amide bands allows the detection of aggregation [47], a major issue for proteins used as pharmaceutical products. It can also be anticipated that the FTIR spectrum could provide information on the level of glycosylation of the protein captured by the sensor, as already demonstrated by direct measurements [16]. The advantage of the measurement through the ATR-FTIR biosensor is that the captured protein can be easily washed from all kinds of soluble excipients. In the absence of this capability, recording the infrared spectrum of a protein requires the removal of the excipients. This step includes tedious procedures and, for some excipients, including the widely used Tween, removal can be almost impossible to achieve.

We developed an ATR-FTIR biosensor able to detect mAbs in a complex culture growth medium and performed a qualitative analysis of protein structure. Importantly, the grafting of various proteins onto the functionalized surface did not result in alteration of the protein structure judging from the well-preserved amide I band shape which is correlated with the protein secondary structure content. The native form of the proteins was therefore maintained during immobilization onto the surface. The successful immobilization of immunoglobulin-binding proteins (Protein A, Protein G, protein A/G) onto the germanium surface allowed the detection of monoclonal antibodies in complex media (culture medium, biopharmaceutical product). The present paper is therefore proof of the principle that the chemistry used results in efficient biosensors able to capture target proteins in a complex environment and to provide high-quality infrared spectra of the captured protein molecules.

## 4. Materials and Methods

### 4.1. Materials

All solvents and reagents were at least of reagent grade quality. Ultrapure water was obtained via a Millipore Milli-Q system (18.2 MΩ.cm) (Merck Millipore, Hoeilaart, Belgium). 1-ethyl-3-[3-(dimethylamino)propyl]carbodiimide (EDC) was purchased from Roth (Carl Roth Gmbh, Karlsruhe, Germany). Additionally, 11-mercaptoundecanoic acid (MUA), N-Hydroxysuccinimide (NHS), bovine serum albumin (BSA), and avidin were obtained from Sigma-Aldrich (Diegem, Belgium). All immunoglobulin-binding proteins (Protein A, G, and AG) were purchased from Prospec-Bio (Ness-Ziona, Israel). Germanium single-crystal triangular prisms (6.8 mm base × 45 mm length and a 45° internal incident angle), shown in Figure 1, were purchased from ACM (Villiers Saint Frédéric, France). These home-designed crystals were described by Goldzstein & coll [27]. Adalimumab (mAb) was produced by a stable CHO-K1 cell line in a fully synthetic CHO-optimized basal medium facilitated by Univercells (Charleroi, Belgium) from a project financed by the Walloon region. The culture medium contained no peptone, fetal bovine serum nor any component of animal origin. In a fed-batch process Adalimumab expression started at day 0 and its concentration increased linearly in the culture medium until day 16 where it reached a concentration of 4.12 mg/mL.

### 4.2. Grafting Procedure

Germanium surfaces were in a first step cleaned to remove any residual molecule used in previous experiments. To do so, the germanium surfaces were cleaned with Superdecontamine (Intersciences, AS, Brussels, Belgium), a lab cleaning detergent solution at pH 13, then successively washed thoroughly with water, ultrapure water, and ethanol. Sonication in acetone was then applied for 15 min. For thiol-SAM functionalization, the cleaned surfaces were immersed in a 50 mM MUA water/isopropanol (IPA) 50/50 solution overnight at 60 °C then rinsed two times in IPA. The activation of the carboxylate function was carried out by immersing the germanium surfaces for 30 min at room temperature in a solution of 50 mM EDC, 50 mM NHS in a 0.1 M MES, 0.5 M NaCl, pH 6.0 buffer. Immunoglobulin-binding proteins immobilization was obtained by incubating the surfaces for 3 h in a 1 mg/mL protein solution in 0.1 M PBS buffer at pH 7.4. Quenching of the unreacted NHS ester was carried out using 1 M ethanolamine pH 9.5 for 5 min. For binding assays, the immunoglobulin-binding proteins coated surfaces were incubated in the mAb solutions for 1 h.

### 4.3. FTIR Spectroscopy

The FTIR measurements were performed at 22 °C on a Bruker Equinox55 FTIR-spectrometer (Bruker Optics GmbH, Ettlingen, Germany) with the software Opus 4.2.37 (Bruker Optics GmbH, Ettlingen, Germany). The FTIR-spectrometer was equipped with a Mercury-Cadmium-Telluride detector, which was cooled down with liquid nitrogen. The spectra were recorded in the ATR mode by using a Golden GateTM ATR accessory (Specac, Orpington, United Kingdom) with an integrated total reflection element composed of a single reflection diamond for reference FTIR spectra or germanium for biosensor analyses. The angle of incidence was 45 degrees. The spectrophotometer was continuously purged with dried air (dew point −40 °C).

Reference FTIR spectra. Spectra of pure molecules were obtained as reference spectra as follows: 0.5 µL of the sample was loaded on the diamond crystal of the ATR device of the FTIR spectrometer and quickly dried with a constant, gentle nitrogen flow. After each spectrum, the crystal was cleaned by gently rubbing its surface with a wet paper tissue. A background was recorded with a clean crystal before the start of the measurements and before every new sample. FTIR spectra were recorded at a scan velocity of 20 kHz between 4000 and 600 cm^−1^ at a resolution of 2 cm^−1^. Each spectrum was obtained by taking an average of 128 scans.

Off-line measurements. A background signal FTIR spectrum of the non-functionalized, clean germanium surface was recorded. After each grafting step, the surface was rinsed thoroughly with the solvent used to deposit the specific molecule on the surface and quickly blown dry with a constant, gentle nitrogen flow before recording the FTIR spectra. 

**Figure 7:**
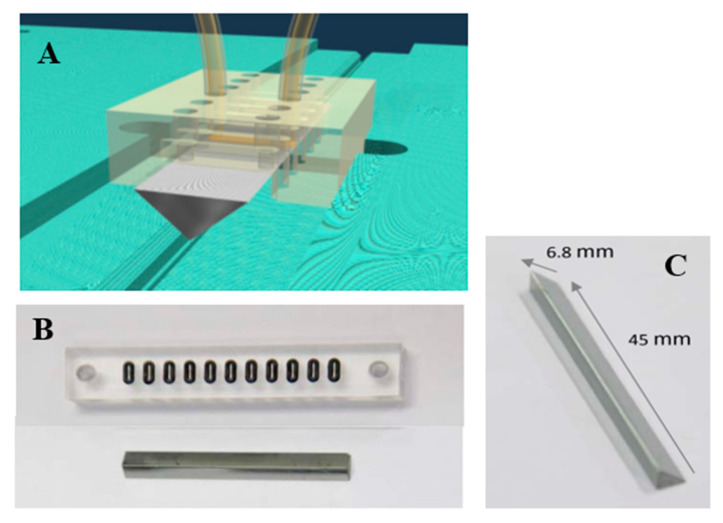
Germanium triangular prism for biosensor. (**A**). Schematic representation of the germanium crystal and of the cover allowing to bring the medium to be analyzed in contact with the crystal. (**B**). Picture of the Plexiglas cover and the O-rings sealing the flushing room with the germanium crystal, 11 channels are available, (**C**). dimensions of the germanium triangular prism.

On-line binding kinetics. Solutions were flowed over the germanium surface through a sealed chamber using a peristaltic pump at a flow rate of 50 µL/min, as depicted in Figure 7A. Due to water absorption in the specific bands of protein in FTIR spectroscopy, deuterated water (D_2_O) was used as a solvent. The germanium IRE and the shape of the measurement cells are depicted in Figure 7B,C respectively.

First, the solution without the protein (buffer) flowed for 5 min. A background FTIR spectrum was recorded. Protein immobilization was carried out by flowing the protein solution for 3 h. An FTIR spectrum was recorded every 3 min (60 spectra per kinetic).

### 4.4. Data Processing

Data were analyzed by using the home-written Kinetics package in Matlab R2007a. All FTIR spectra were preprocessed as follows. The water vapor contribution was subtracted as described previously [48,49] with 1956–1935 cm^−1^ as reference peak. The spectra were then baseline-corrected.

Straight lines were interpolated between spectrum absorbance at the following wavenumbers: 

3700, 3000, 2800, 1760, 1658, 1500, 1385, 1140, 1000, and 835 cm^−1^ for MUA grafting.

3700, 3000, 2800, 2000, 1850, 1700, 1240, 1120, 1016, and 950 cm^−1^ for EDC/NHS activation.

3700, 3000, 2800, 2000, 1830, 1760, 1700, 1577, 1464, 1368, and 950 cm^−1^ for protein grafting.

3700, 3000, 2800, 1718, 1476, 1179, 965, and 865 cm^−1^ for antibody binding.

They were then subtracted from the spectrum. A Gaussian apodization to 8 cm^−1^ was applied. For the analysis of antibody binding, an area normalization was applied on the amide bands (between 1718 and 1476 cm^−1^).

### 4.5. Immunoglobulin Cleanup

Micro Bio-Spin^TM^ P-6 size exclusion spin columns (Bio-Rad #7326221, Tris buffer, sample volume 10–75µL, 6 kDa MW limit) (Bio-Rad, Temse, Belgium) have been used to remove buffer and salt molecules that can interfere with the FTIR reference spectra of the antibody. The buffer was exchanged by 0.9% NaCl.

### 4.6. Antibody Purification

After elimination of the cells by filtration, the antibody has been captured using protein A affinity chromatography and eluted at low pH, after which held at low pH for 45 min. The antibody has been further purified by anion exchange and mixed-mode chromatography.

## 5. Conclusions

For ATR-FTIR biosensors, the first step of sensor building is to achieve a reproducible and stable layer of grafted molecules which bear a second chemical function amenable to some kind of chemistry resulting in the attachment of receptors. The approach described in the present paper is particularly simple and results in very reproducible coverage of the germanium surface. We demonstrated that it can be an efficient basis for building biosensors by attaching protein A, protein G, and other proteins which, in turn, could bind antibodies.

## Data Availability

Data are available from request to the author OS.

## Supplementary Materials

Supplementary Figures labelled from S1 to S11 can be downloaded at: https://www.mdpi.com/article/10.3390/ijms231911895/s1 in the “Supplementary Information.pdf file”.

## Abbreviations

ATR	attenuated total reflection
BSA	bovine serum albumin
CQA	critical quality attribute
EDC	1-ethyl-3-[3-(dimethylamino)propyl]carbodiimide
FTIR	Fourier-transform infrared
IPA	isopropyl alcohol
IRE	internal reflection element
mAb	monoclonal antibody
MUA	11-mercaptoundecanoic acid
NHS	N-hydroxysuccinimide
PBS	Phosphate Buffered Saline
SAM	self-assembled monolayer
