# Supplementary material for: ATR-FTIR Biosensors for Antibody Detection and Analysis
Source: Int J Mol Sci. 2022 Oct 7;23(19):11895. doi: 10.3390/ijms231911895 (PMC9570191; doi:10.3390/ijms231911895)
Supplement: Supplementary file 1 [file ijms-23-11895-s001.zip › ijms-1926205-Supplementary Information.pdf]

Supplementary materials for

ATR-FTIR biosensors for antibody detection

Olivier Suys, Allison Derenne and Erik Goormaghtigh

Center for Structural Biology and Bioinformatics, Laboratory for the Structure and Function of Biological Membranes, Campus Plaine CP206/02; Université Libre de Bruxelles, B1050 Brussels, Belgium

Contents

Figure S1: grafting of 11-mercaptoundecanoic acid

Figure S2: grafting deuterated d_{25} dodecanethiol

Figure S3: NHS ester formation

Figure S4: BSA immobilization

Figure S5: BSA immobilization kinetic correlation

Figure S6: Avidin immobilization

Figure S8: Protein G immobilization kinetics

Figure S9: Structural integrity after grafting

Figure S10: mAb grafting control

Figure S11: mAb grafting control

Figure S1: Grafting of 11-mercaptoundecanoic acid

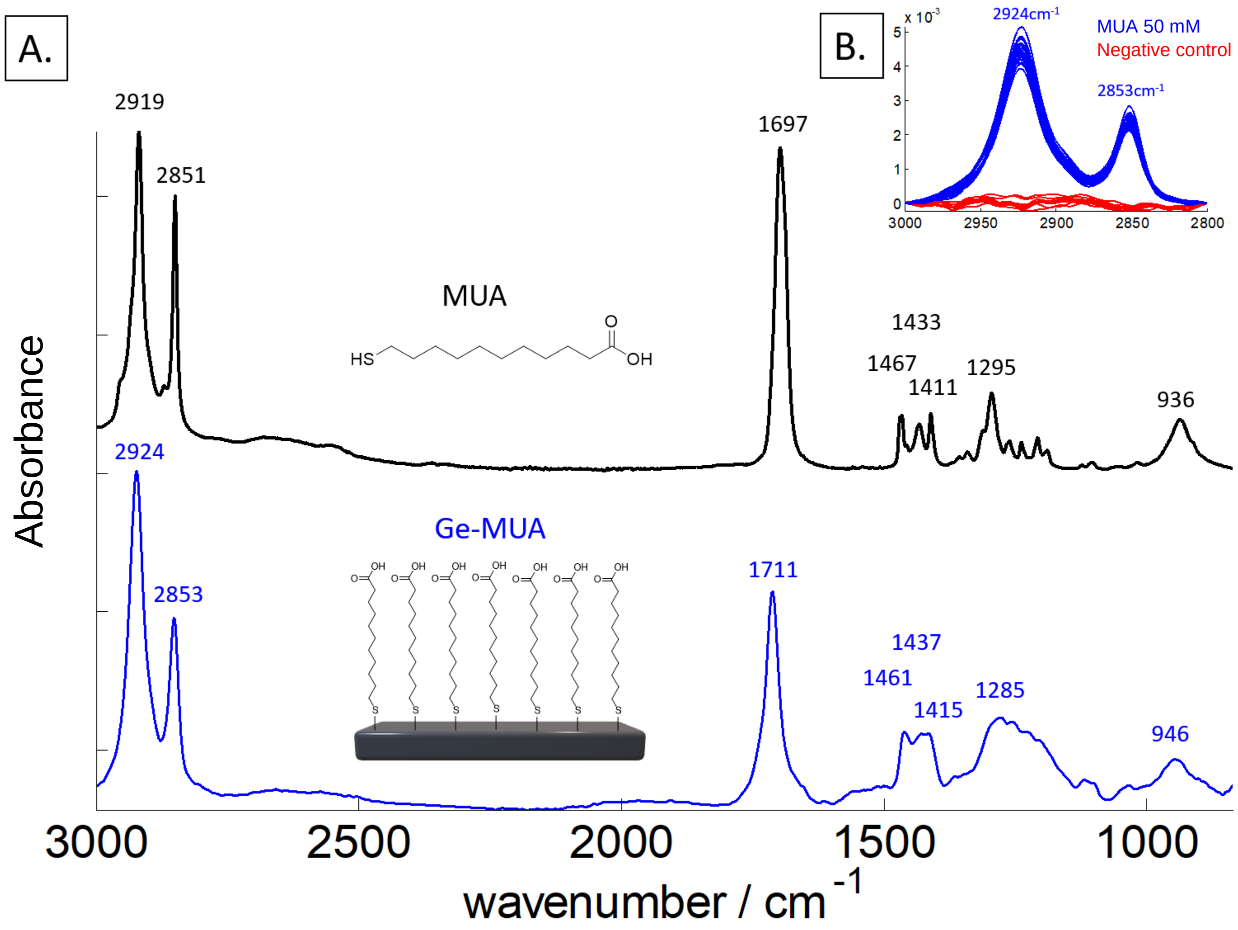

Figure S1: A. Ge oxide removal and 11-mercaptoundecanoic acid (MUA) SAM functionalization using 1:1 IPA-water 50mM thiol solution heated at 60°C (blue line). The FTIR spectrum of the 11-mercaptoundecanoic acid grafted onto the surface is characterized by three well-defined bands: $\nu_{as}(CH_2)$ at 2924cm^{-1}, $\nu_s(CH_2)$ at 2853cm^{-1} and ν(C=O) at 1711 cm^{-1}. The black line is the spectrum of pure MUA in IPA dried onto the diamond crystal. Spectra have been vertically offset for the sake of the clarity. B. Ge crystals immerged in IPA without MUA (negative control) show no specific bands. The inset reports the results in the 3000-2800 cm^{-1} spectral range for 20 repetitions for MUA binding and 10 repetitions for the control.

Figure S2: Grafting of deuterated d_{25} dodecanethiol

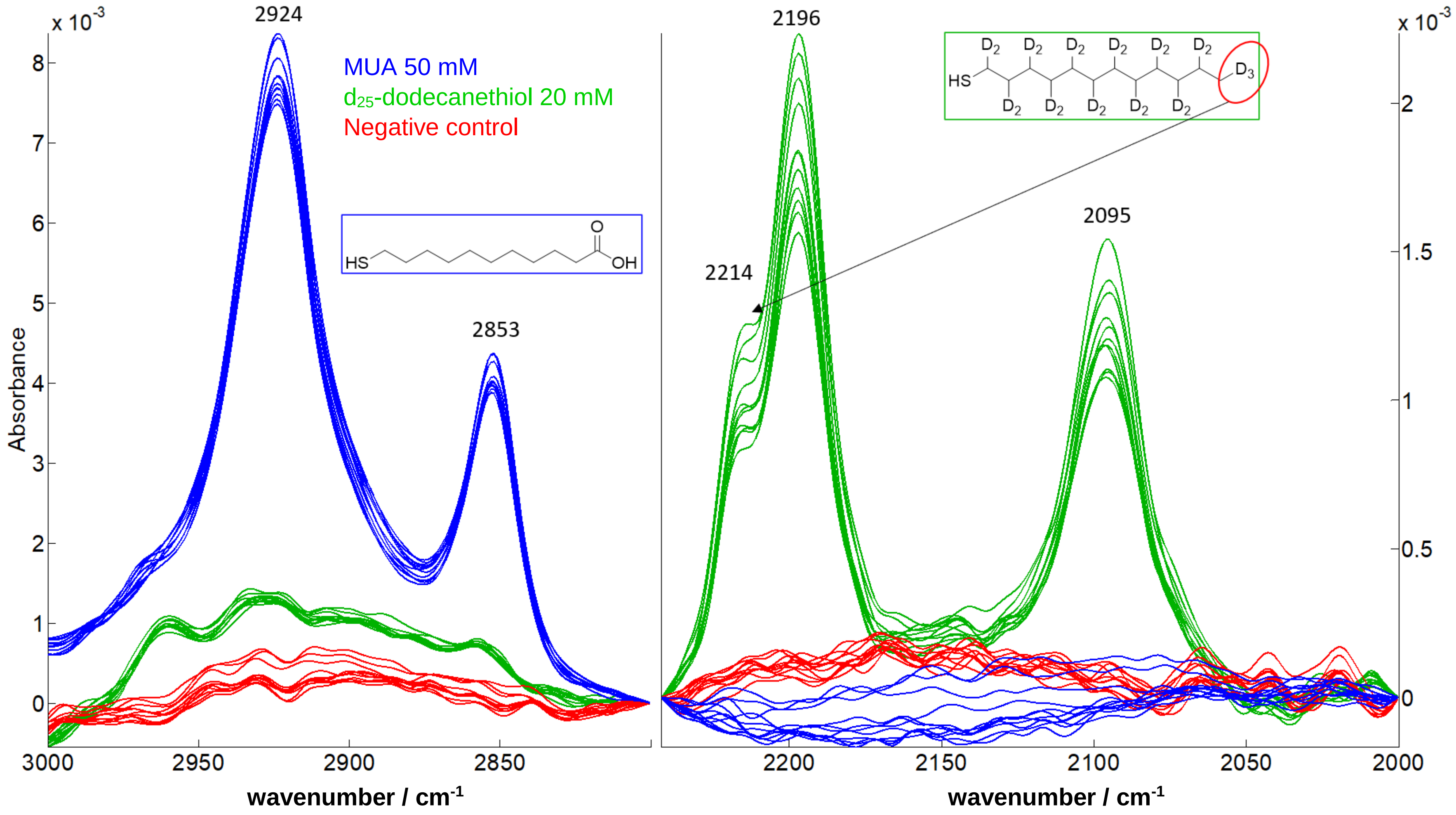

Figure S2: Grafting of 50mM MUA (blue line), 20 mM deuterated dodecanethiol (green line) and control (red line). The grafting of fully deuterated MUA (green line) is characterized by the bands ν_{as} (C^2H_2) at 2196 cm^{-1}, ν_s (C^2H_2) at 2095 cm^{-1} and ν_{as}(C^2H_3) at 2214 cm^{-1}. Ten independent spectra are reported for each condition.

Figure S3: NHS ester formation

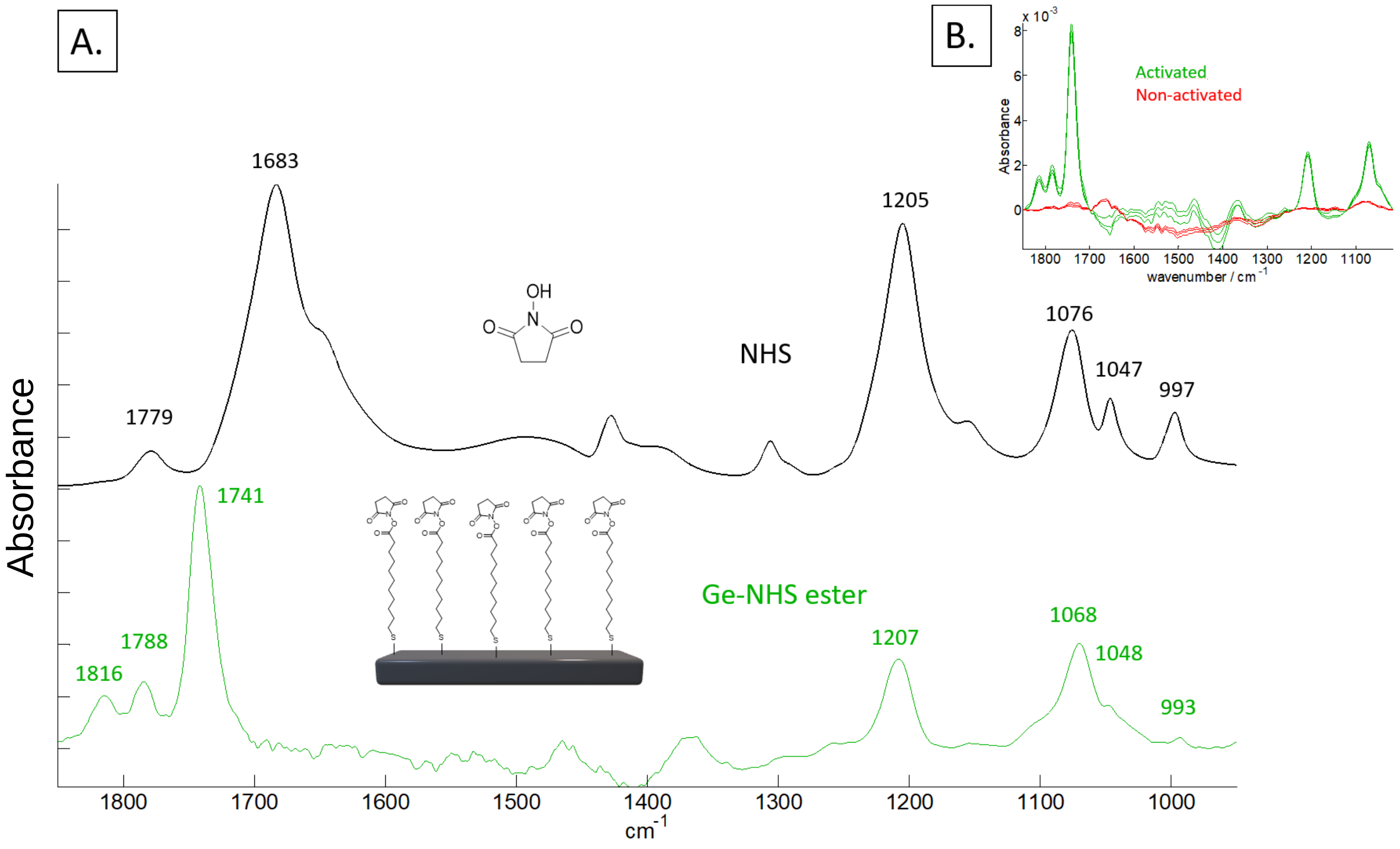

Figure S3: A. Activation of the carboxyl group of MUA using EDC/NHS. The black line is the spectrum of pure NHS in water dried onto the diamond IRE. The green line is the carboxyl of Ge grafted-MUA transformed into an NHS ester function. The band at 1683 cm^{-1} of the black line corresponds to the vibrations of the ketone's groups of the NHS while the band at 1741 cm^{-1} of the green line corresponds to the vibration of the ester bond. Two additional well-defined bands of the ester bond are observable at 1816 cm^{-1} and 1788 cm^{-1} and two bands specific to NHS are present at 1207 cm^{-1} and 1068 cm^{-1}. Spectra have been vertically offset for the sake of the clarity. B. Comparison of FTIR spectra of activated and non-activated MUA. For non-activated MUA, the Ge grafted-MUA crystals were immerged in water without EDC/NHS (negative control). Spectra are presented for 4 independent experiments.

Figure S4: BSA immobilization

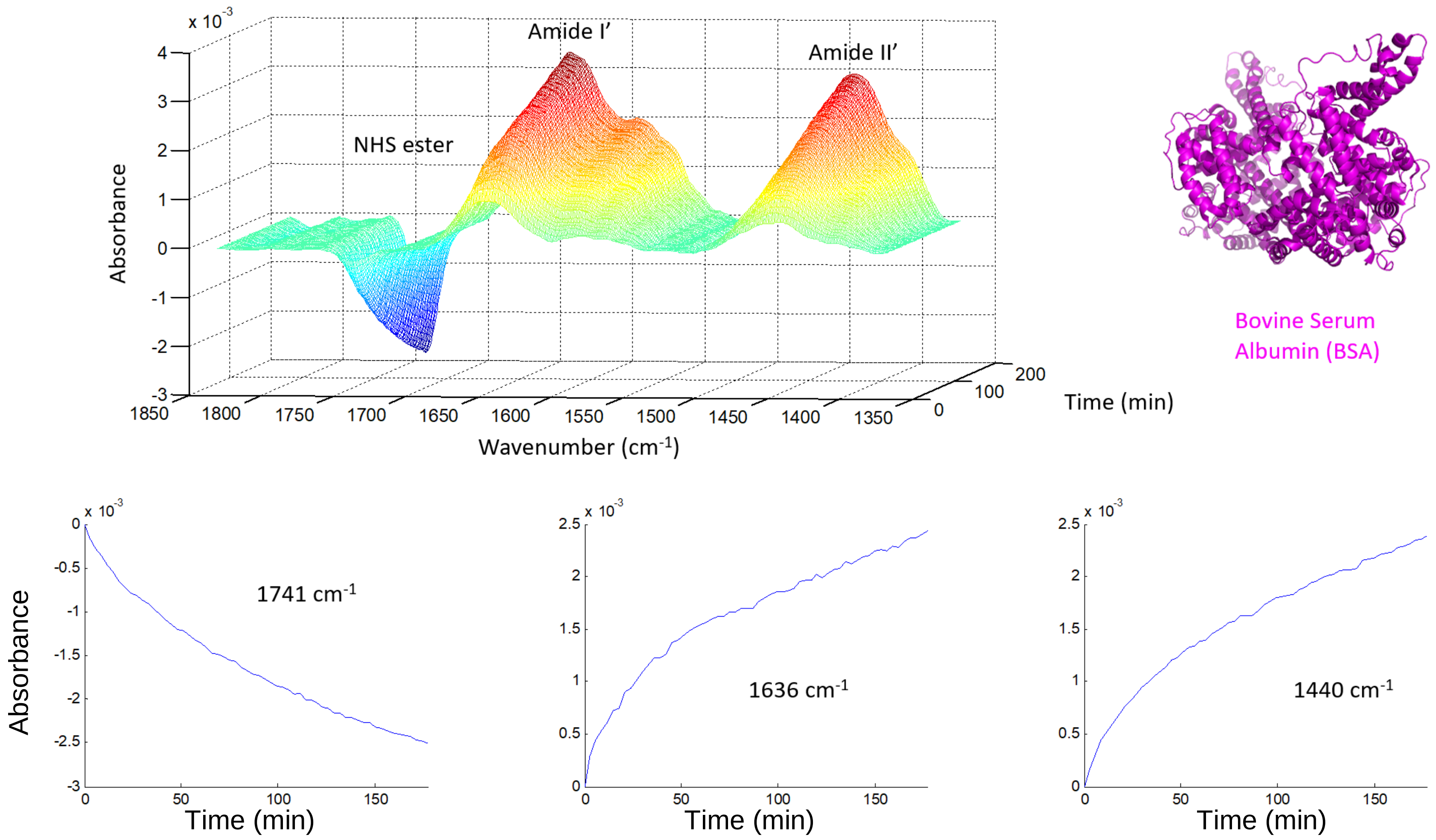

Figure S4: Grafting kinetics of bovine serum albumin on the activated germanium surface monitored by ATR-FTIR spectroscopy. The color gradient indicates the absorbance intensity. As the NHS ester band at 1741 cm^{-1} disappears (in blue), amide bands at 1636 cm^{-1} and 1440 cm^{-1} appear (in red) over time. Duration of the kinetics is 3 hours, and a spectrum is recorded every 3 min (60 spectra in total). Evolution of the absorbance of the three specific wavenumbers over time (min) is shown below.

Figure S5: BSA immobilization kinetic correlation

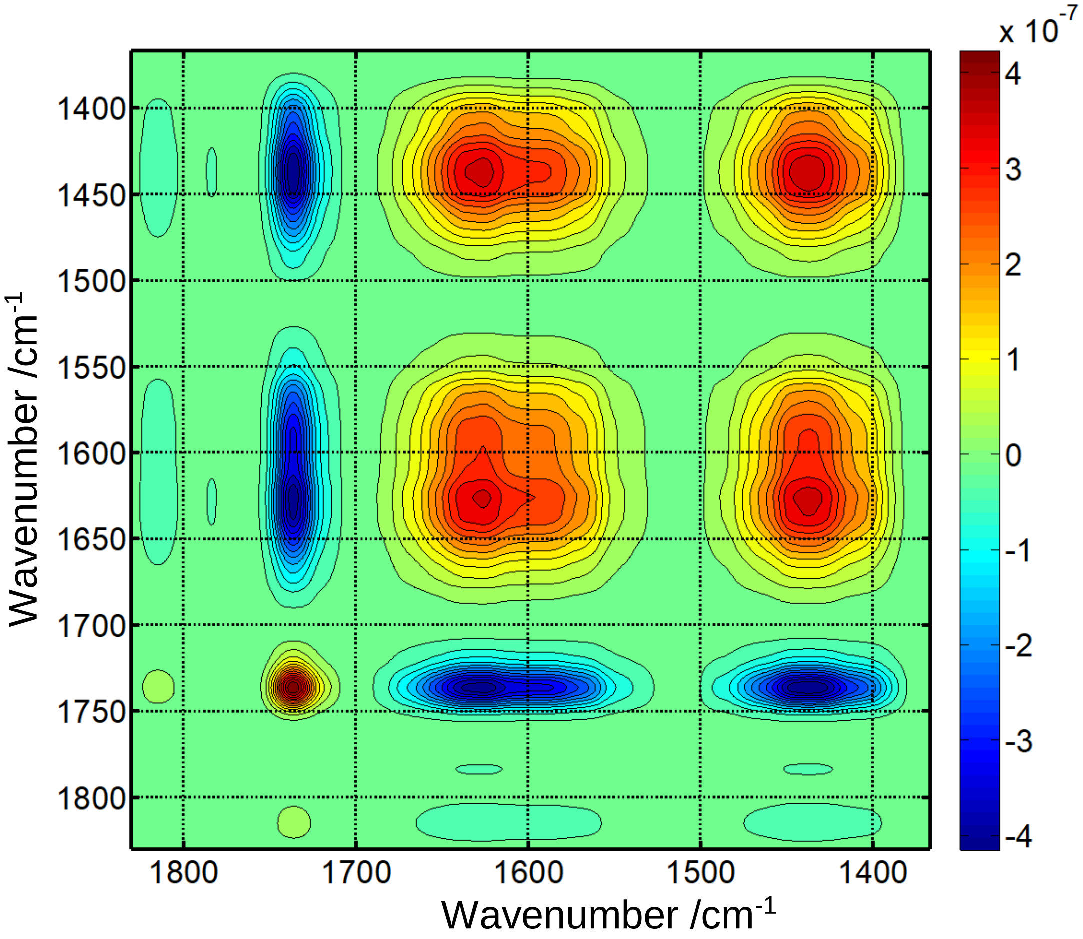

Figure S5: Magnitude of the covariance between the different wavenumbers during the grafting of BSA. The disappearance of the NHS ester at 1741cm^{-1} is significantly and negatively correlated with the appearance of the amide bands at 1636 cm^{-1} and 1640 cm^{-1}. Correlation coefficient between 1741 and 1636: -0.993, corresponding p-value: 3.3 10^{-55}. Correlation coefficient between 1741 and 1440 cm^{-1}: -0.998, corresponding p-value: 4.96 10^{-72}. Correlation coefficient between 1636 and 1440 cm^{-1}: +0.997, corresponding p-value: 3.610^{-68}.

Figure S6: Avidin immobilization

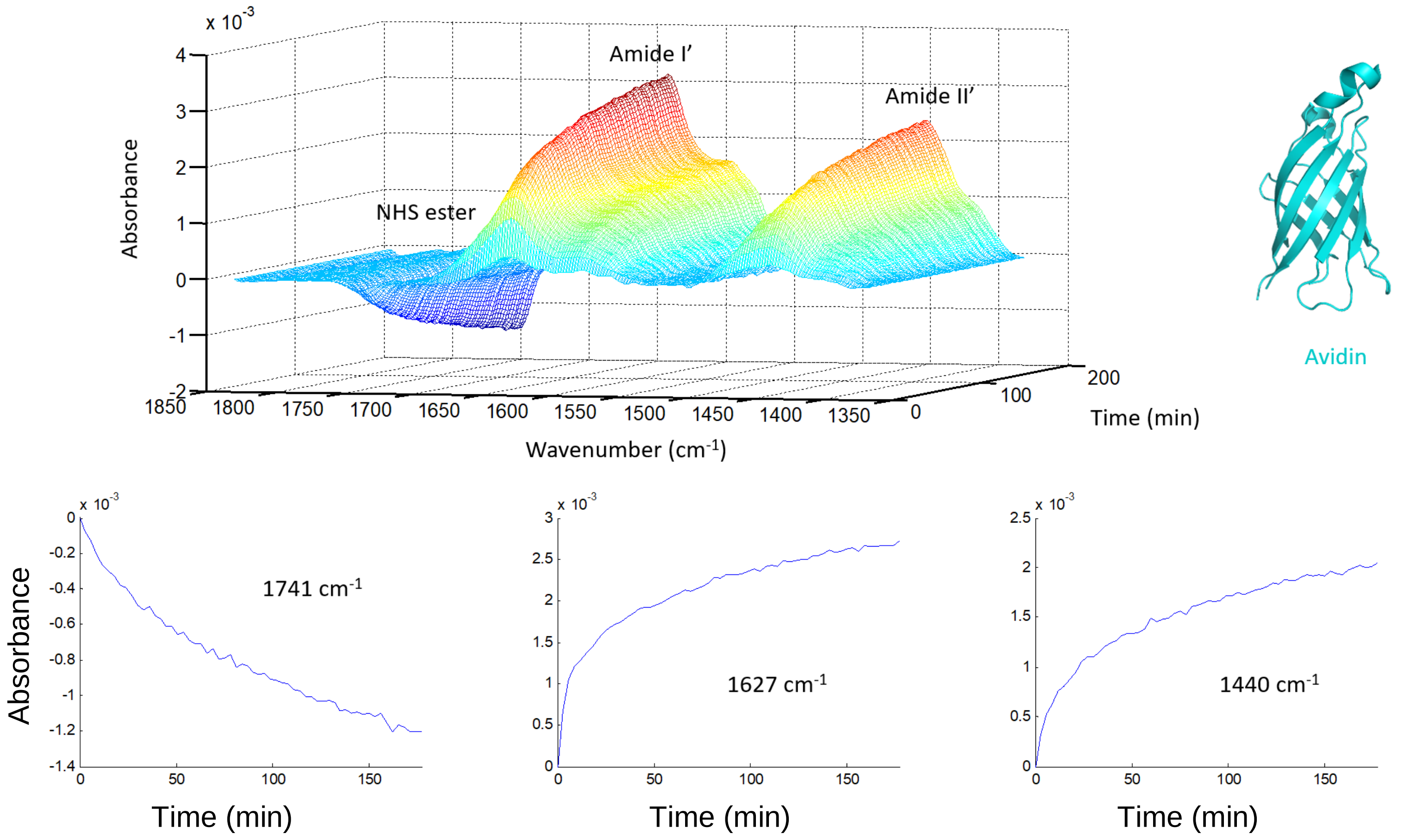

Figure S6: Grafting kinetics of avidin on the activated germanium surface monitored by ATR-FTIR spectroscopy. The color gradient indicates the absorbance intensity. As the NHS ester band at 1741 cm^{-1} disappear in blue, amide bands at 1627 cm^{-1} and 1440 cm^{-1} appear in red over time. Duration of the kinetics is 3 hours, and a spectrum is recorded every 3 min (60 spectra in total). Evolution of the absorbance of the three specific wavenumbers over time (min) is shown below.

Figure S7: avidin immobilization kinetic correlation

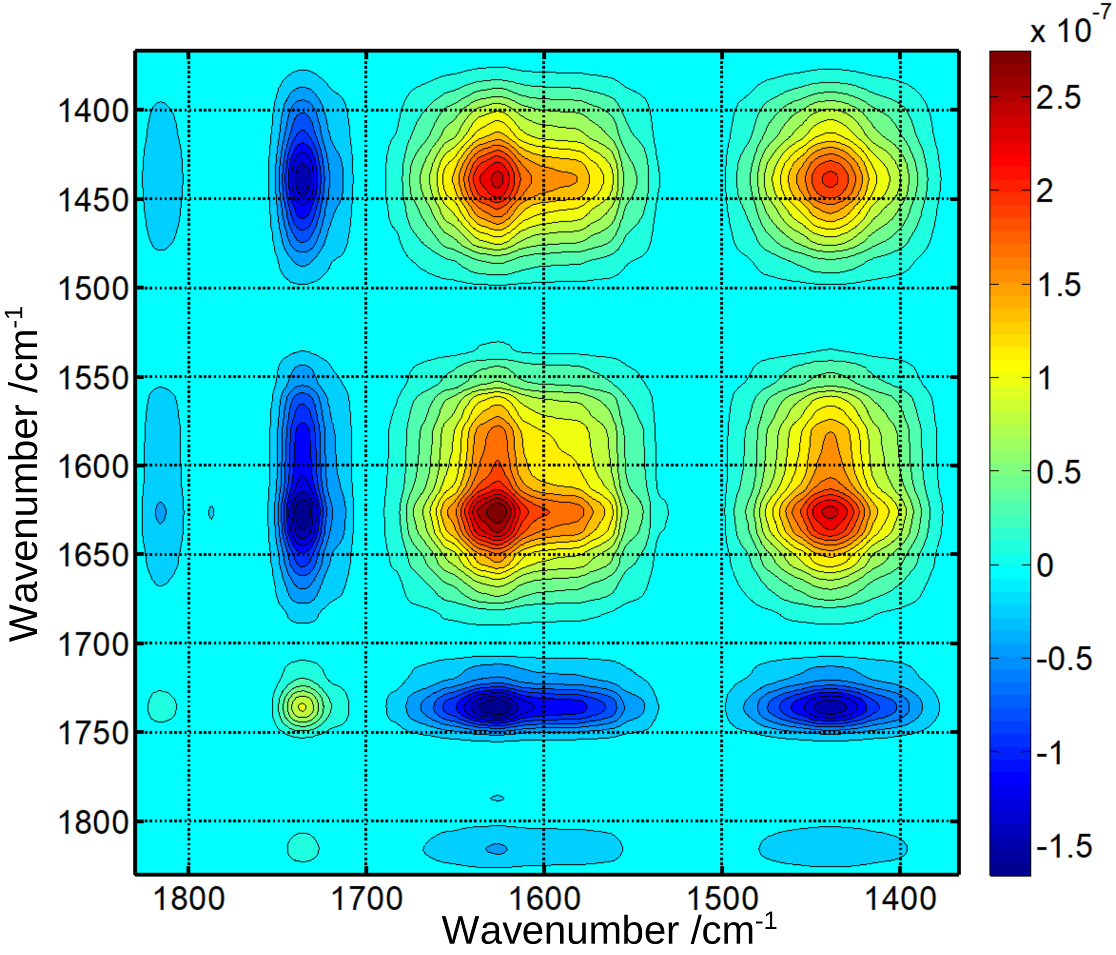

Figure S7: Magnitude of the covariance between the different wavenumbers during the grafting of avidin. The disappearance of the NHS ester at 1741 cm^{-1} is significantly and negatively correlated with the appearance of the amide bands at 1627 cm^{-1} and 1640 cm^{-1}. Correlation coefficient between 1741 cm^{-1} and 1627 cm^{-1}: -0.967, corresponding p-value: 3.2 10^{-36}. Correlation coefficient between 1741 cm^{-1} and 1440 cm^{-1}: -0.986, corresponding p-value: 1.3$10^{-46}$. Correlation coefficient between 1627 cm^{-1} and 1440 cm^{-1}: +0.994, corresponding p-value: 6.3$10^{-54}$

Figure S8: Protein G immobilization

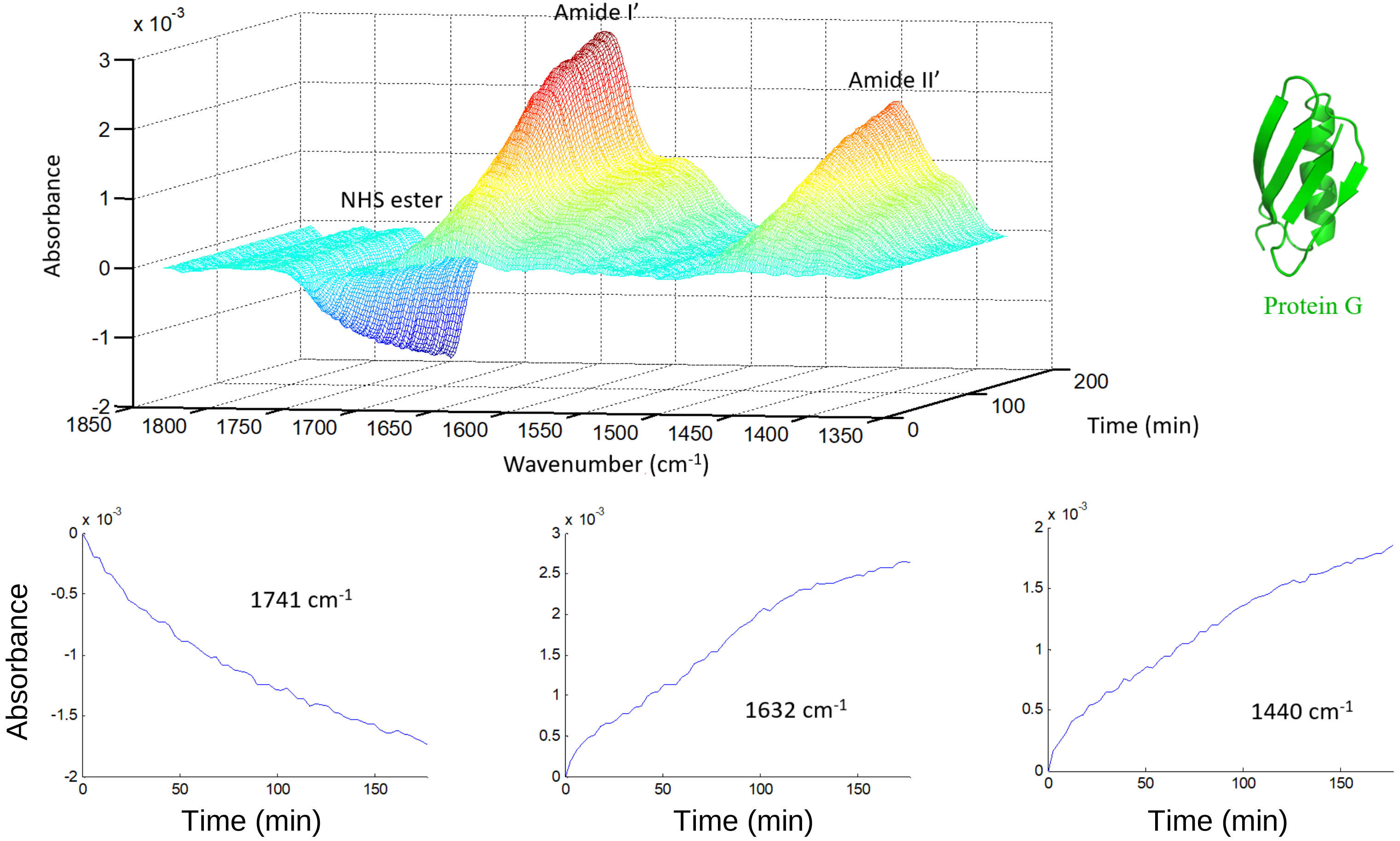

Figure S8: Grafting kinetics of protein G on the activated germanium surface monitored by ATR-FTIR spectroscopy. The color gradient indicates the absorbance intensity. As the NHS ester band at 1741 cm^{-1} disappear in blue, amide bands at 1632 cm^{-1} and 1440 cm^{-1} appear in red over time. Duration of the kinetics is 3 hours, and a spectrum is recorded every 3 min (60 spectra in total). Evolution of the absorbance of the three specific wavenumbers over time (min) is shown below.

Figure S9: Preservation of the structural integrity

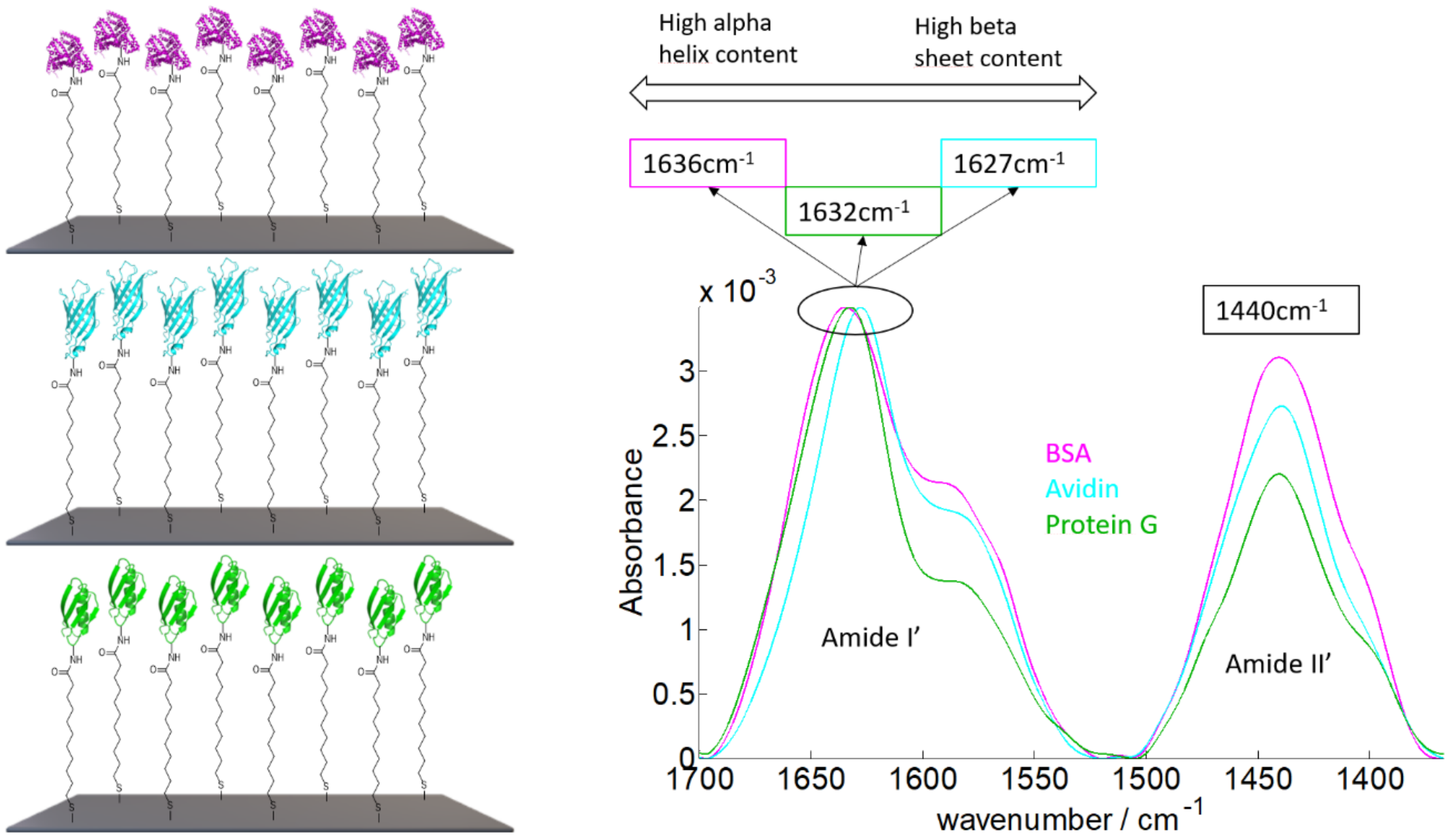

Figure S9: FTIR spectra of the immobilization of three very different proteins in terms of secondary structure. Bovin Serum Albumin (purple) is mainly composed of alpha-helices, avidin (blue) contains mainly beta-sheets and the immunoglobulin-binding protein, protein G (green) contains a mix of both. The wavelength of the Amide I' band of each protein is consistent with its secondary structure content.

Figure S10: mAb grafting control

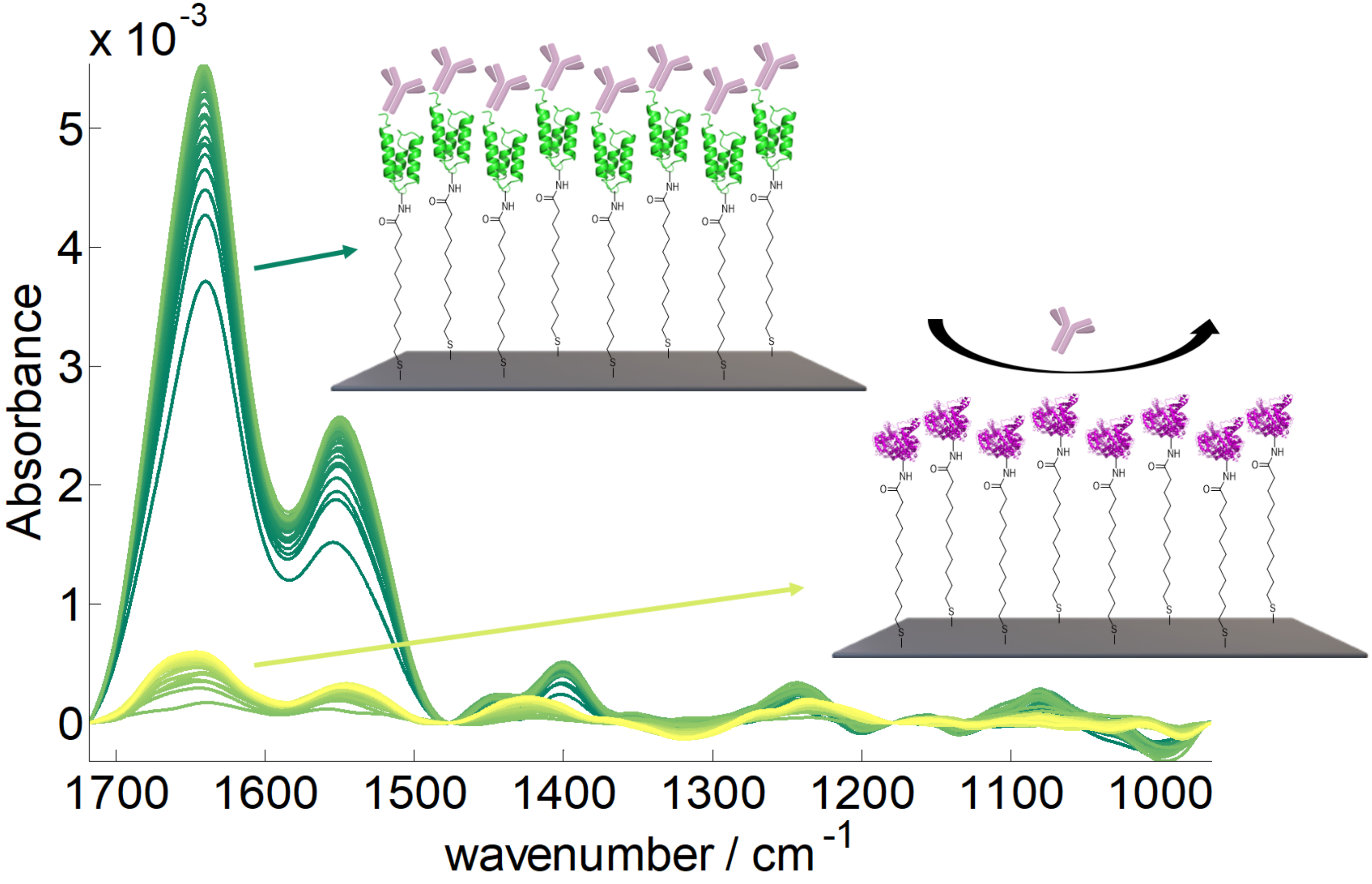

Figure S10: Specificity of antibody binding from a complex culture medium onto the developed biosensor. The series of spectra (in green) depicts the binding of antibodies present in a culture medium onto a protein A coated germanium surface as a function of the time. On a BSA coated surface (series of spectra in yellow) no significant binding of antibodies occurred.

Figure S11: mAb grafting control

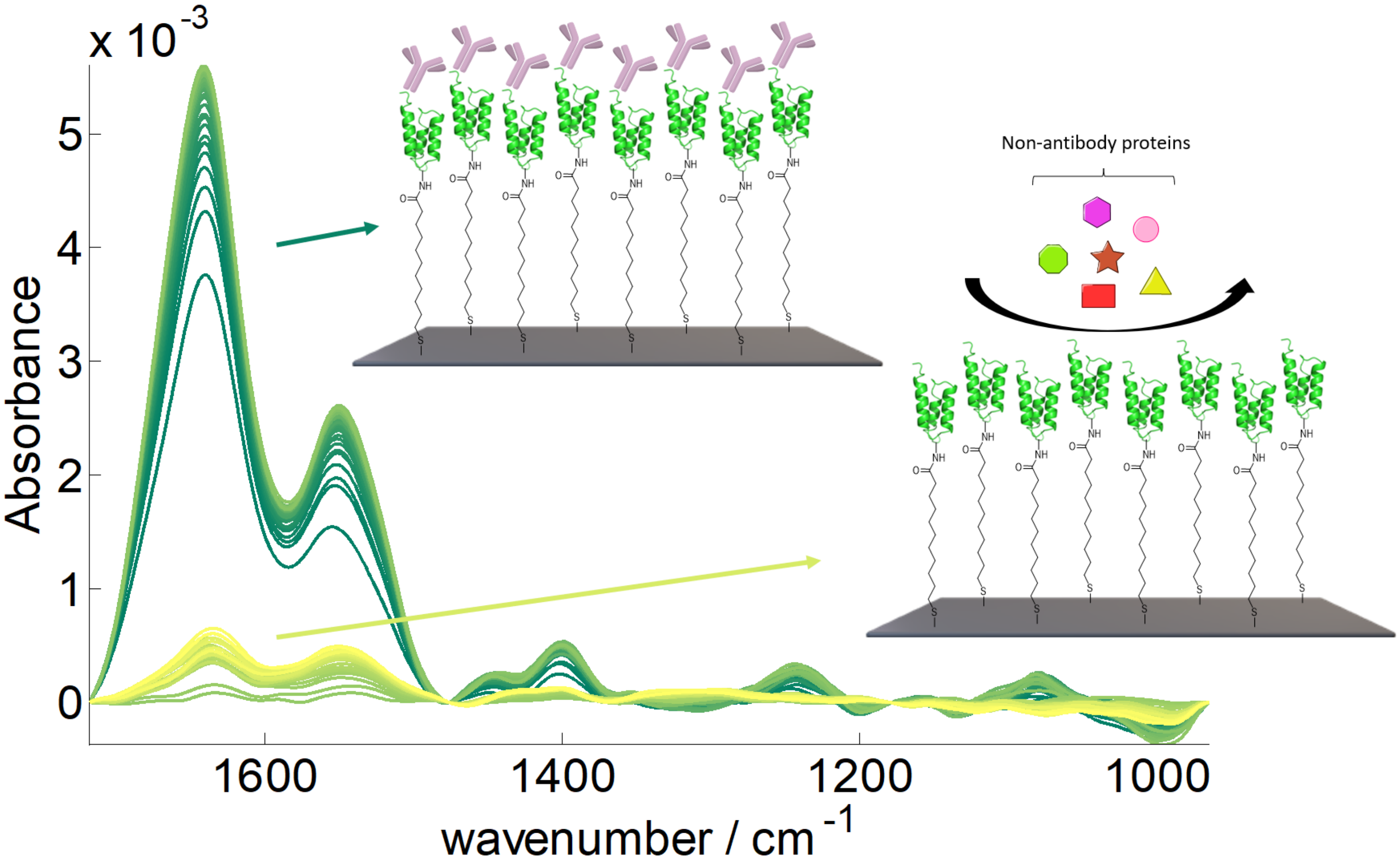

Figure S11: Specificity of antibody binding from a complex culture medium onto the developed biosensor. The series of spectra in green reports the binding of antibodies from a culture medium at day 16 of growth onto the protein A coated germanium surface. Spectra are the same as those reported in Figure S10. When a culture medium at day 0 containing no antibody (series of spectra in yellow) was used, no significant binding of antibodies occurred.
